# Repurposing homoharringtonine for thyroid cancer treatment through TIMP1/FAK/PI3K/AKT signaling pathway

**DOI:** 10.1016/j.isci.2024.109829

**Published:** 2024-04-26

**Authors:** Chuang Xi, Guoqiang Zhang, Nan Sun, Mengyue Liu, Nianting Ju, Chentian Shen, Hongjun Song, Quanyong Luo, Zhongling Qiu

**Affiliations:** 1 Department of Nuclear Medicine, Shanghai Sixth People’s Hospital Affiliated to Shanghai Jiao Tong University School of Medicine, Shanghai 200233, China

**Keywords:** Phytopharmacy, Molecular biology, Cell biology, Cancer

## Abstract

Homoharringtonine (HHT), an alkaloid isolated from *Cephalotaxus*, is an effective anti-leukemia agent and exhibits inhibitory effects in various solid tumors. However, the impacts of HHT treatment on thyroid cancer (TC) remain unclear. Our findings demonstrated that HHT exhibited remarkable anti-TC activity that involved inhibiting cell proliferation, invasion, and migration, as well as inducing apoptosis. Proteomics analysis revealed that the expression of the tissue inhibitor of metalloproteinase 1 (TIMP1) was downregulated in TC cells after HHT treatment. TIMP1 overexpression promoted TC progression and partially reversed the anti-TC effects of HHT, while TIMP1 downregulation inhibited TC progression and enhanced the anti-TC effects of HHT. Furthermore, TIMP1 re-expression attenuated the enhancement of anti-TC effects of HHT induced by TIMP1 knockdown. Mechanistically, HHT exerted anti-TC effects by downregulating TIMP1 expression and then inactivating the FAK/PI3K/AKT signaling pathway. Taken together, our study demonstrated that HHT could inhibit TC progression by inhibiting the TIMP1/FAK/PI3K/AKT signaling pathway.

## Highlights

• HHT exhibits remarkable anti-tumor effect in thyroid cancer
• The anti-thyroid cancer effect of HHT is dependent on TIMP1 downregulation
• HHT inhibits thyroid cancer progression by inactivating TIMP1/FAK/PI3K/AKT axis

## Graphical abstract

**Figure:**
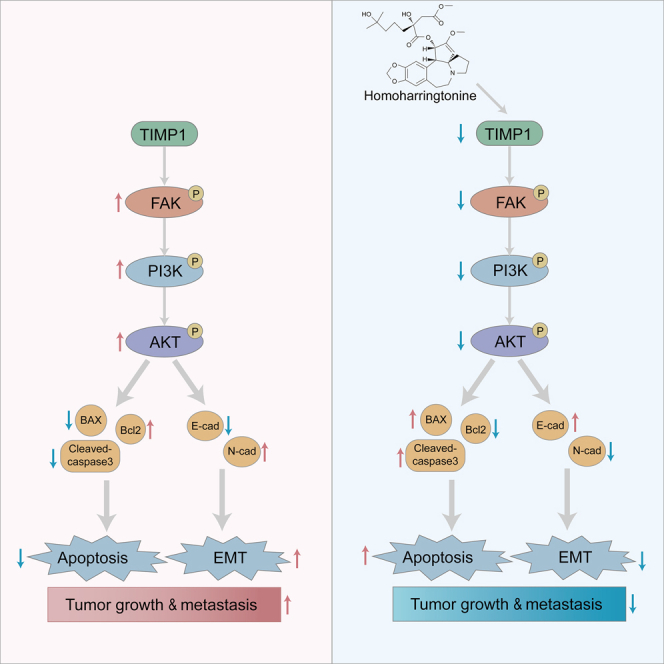

## Introduction

Thyroid cancer (TC) is the most prevalent endocrine malignancy with a remarkably increasing incidence worldwide in recent years.^1^^,^^2^ TC originating from follicular cells accounts for over 90% of diagnosed TC cases and is histologically divided into differentiated TC (DTC), poor differentiated TC (PDTC), and anaplastic TC (ATC).^3^ DTC is further divided into papillary TC (PTC) and follicular TC.^4^ The majority of DTC patients have excellent prognoses following conventional therapies consisting of surgery, radioiodine therapy, and thyroid stimulating hormone-suppressed treatment. Nevertheless, some patients suffer from aggressive disease and eventually progress to PDTC or ATC, which exhibit resistance to conventional therapies and are associated with poor prognoses.^5^ Consequently, treatment for aggressive diseases is a particularly difficult challenge in the management of TC patients.

Repurposing the US Food and Drug Administration (FDA)-approved drugs for new indications could be a feasible approach to overcome current cancer treatment obstacles.^6^ Homoharringtonine (HHT), a natural alkaloid isolated from *Cephalotaxus*, has been used as an anti-leukemia drug since the 1970s.^7^ Clinically, HHT effectively inhibits hematological malignancies and has received approval from the FDA for chronic myeloid leukemia treatment. HHT also shows significant inhibitory effects in various solid tumors, such as breast, liver, and bladder cancers.^8^^,^^9^^,^^10^^,^^11^ Mechanistically, HHT exerts anti-tumor activity by preventing aminoacyl-tRNA binding to ribosomes during mRNA translation, resulting in protein synthesis arrest.^12^^,^^13^ However, to our knowledge, whether HHT has anti-tumor potential in TC and the underlying molecular mechanisms have not been studied.

In the present study, we demonstrated that HHT exhibits remarkable anti-tumor effects on TC *in vitro* and *in vivo*. Mechanistically, HHT results in the downregulation of the tissue inhibitor of metalloproteinase 1 (TIMP1), and then exerts the anti-TC effects by inactivating the TIMP1/FAK/PI3K/AKT signaling pathway.

## Results

### HHT suppresses TC progression *in vitro*

**Figure 1:**
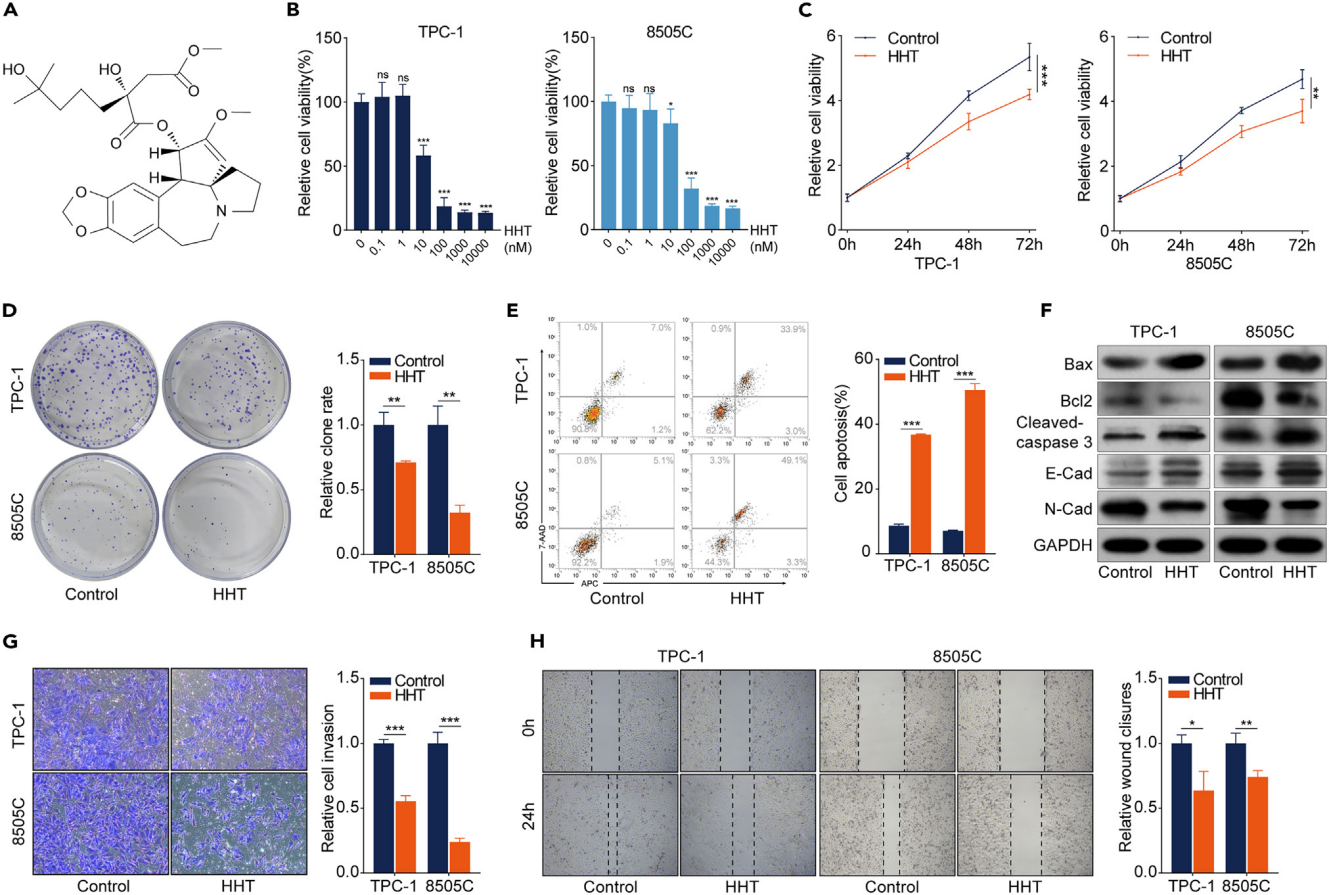
HHT suppresses TC progression *in vitro* (A) The chemical structure of HHT. (B) CCK-8 assay was performed to detect cell viability of TPC-1 and 8505C cells treated with increased gradients of HHT for 48 h. (C) CCK-8 assay was performed to detect cell viability of TPC-1 and 8505C cells with HHT treatment for 24, 48, and 72 h. (D) Colony formation assay was performed to evaluate the clonogenic ability of TPC-1 and 8505C cells with HHT treatment for 14 days. (E) The effect of HHT on the apoptosis of TPC-1 and 8505C cells was detected by flow cytometry. (F) Western blotting analysis of the expression of Bax, Bcl2, cleaved caspase-3, E-cadherin, and N-cadherin in TPC-1 and 8505C cells after HHT treatment for 48 h. GAPDH was used as the control. (G) Transwell assay was used to measure the invasion ability of TPC-1 and 8505C cells after HHT treatment for 24 h. (H) The wound healing assay was performed to detect the migration rate of TPC-1 and 8505C cells after HHT treatment for 24 h. Data are presented as mean ± SD. ns, no significance, ∗*p* < 0.05, ∗∗*p* < 0.01, ∗∗∗*p* < 0.001.

To investigate the therapeutic potential of HHT in TC, a series of biological experiments were conducted. The HHT chemical structure is depicted in Figure 1A. The CCK-8 assay showed that after being treated with an increased concentration of HHT for 48 h, the viability of TPC-1 and 8505C cells was remarkably inhibited in a dose-dependent manner (Figure 1B). The half-maximal inhibitory concentration values of TPC-1 and 8505C were 19 ± 0.8 and 56 ± 4.6 nM, respectively (Figure S1). Therefore, we used 20 nM HHT to treat TPC-1 cells and 50 nM HHT to treat 8505C cells in subsequent research. The CCK-8 assay also showed that HHT significantly decreased cell viability time dependently (Figure 1C). The colony formation assay demonstrated that HHT significantly attenuated the clonogenic ability of TPC-1 and 8505C cells (Figure 1D). An underlying anti-tumor mechanism of HHT is inducing cell apoptosis^14^^,^^15^; flow cytometry analysis was conducted to investigate the effects of HHT on apoptosis of TC cells and the results suggested that the rate of apoptotic cells was dramatically increased in TPC-1 and 8505C cells after HHT treatment (Figure 1E). This pro-apoptotic role of HHT was further confirmed by detecting the expression of pro-apoptotic proteins (Bax and cleaved caspase-3) and anti-apoptotic protein (Bcl2) (Figures 1F and S2). Subsequently, results from the Transwell assay and wound healing assay demonstrated that HHT significantly impaired the invasion and migration abilities of TPC-1 and 8505C cells (Figures 1G and 1H), which was accompanied by upregulation of E-cadherin and downregulation of N-cadherin (Figures 1F and S2). HHT also robustly exhibited anti-tumor effects on other types of TC cell lines, BCPAP and FTC133 (Figure S3). Radioiodine therapy is a cornerstone for the treatment of unresectable and/or metastatic DTC, which is dependent on sufficient sodium/iodide symporter (NIS) expression and its localization to the plasma membrane of TC cells.^16^^,^^17^ Therefore, we preliminarily detected the NIS expression in TC cells after HHT treatment and found that HHT significantly enhanced the NIS expression at the mRNA and protein levels (Figure S4). Taken together, these results prove that HHT exhibits broad-spectrum anti-TC functions, which involve inhibition of cell proliferation, invasion, and migration, as well as induction of apoptosis.

### The anti-TC effects of HHT are associated with the FAK/PI3K/AKT signaling pathway and TIMP1 downregulation

**Figure 2:**
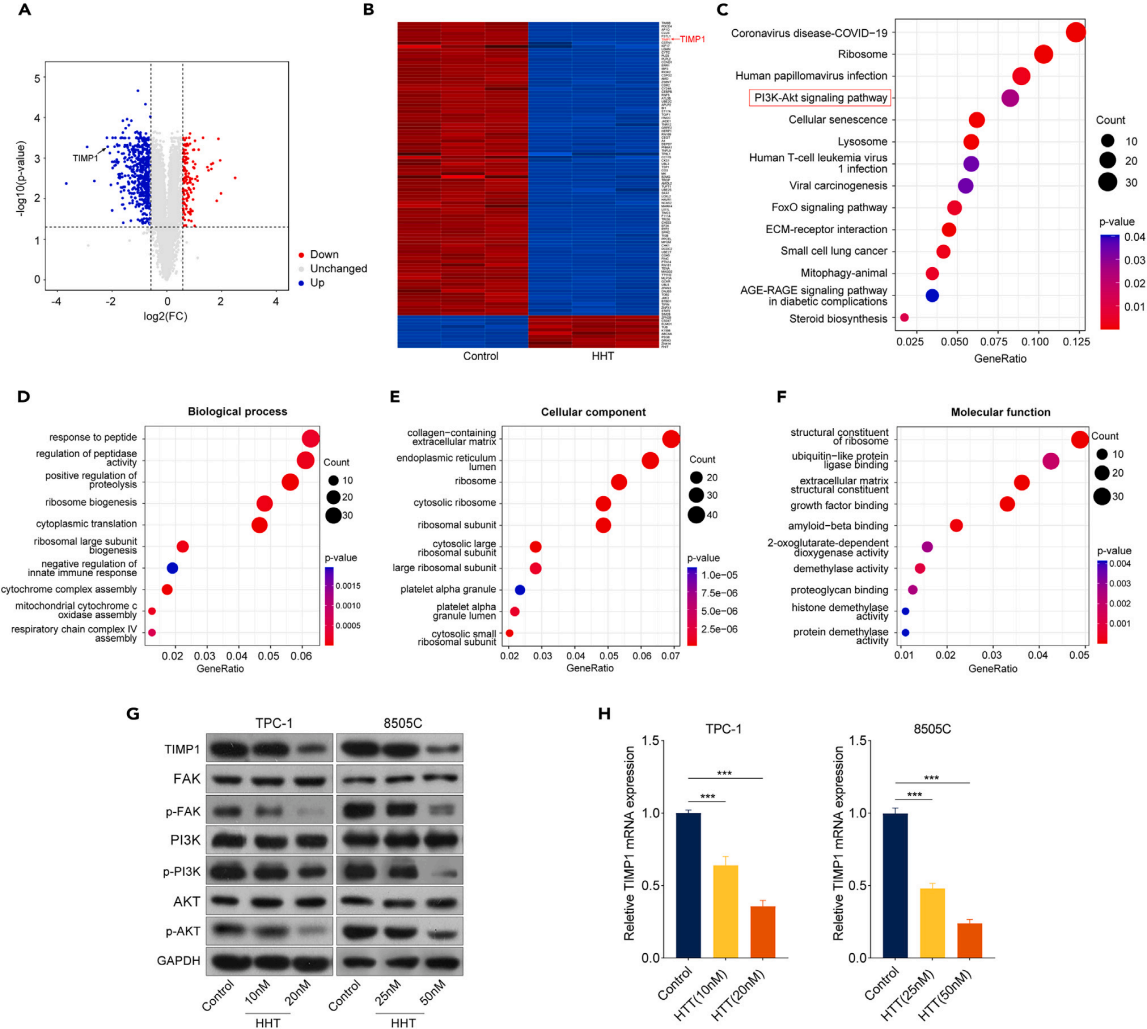
The anti-TC effects of HHT are associated with FAK/PI3K/AKT signaling pathway and TIMP1 downregulation (A) The volcano plot showed the 787 differentially expressed proteins in TPC-1 cells between HHT-treated (20 nM, 48 h) and control groups (fold change ≥1.5 or ≤ −1.5 and *p* values <0.05). (B) The heatmap showed the top 100 differentially expressed proteins. (C) KEGG analysis of the differentially expressed proteins. (D–F) GO analysis of the differentially expressed proteins. (G) Western blotting analysis of the protein expression of TIMP1, FAK, p-FAK, PI3K, p-PI3K, AKT, and p-AKT in TPC-1 and 8505C cells after HHT treatment for 48 h. GAPDH was used as the control. (H) RT-qPCR analysis of the mRNA expression of TIMP1 in TPC-1 and 8505C cells after HHT treatment for 48 h. GAPDH was used as the control. Data are presented as mean ± SD. ∗∗∗*p* < 0.001.

To elucidate the underlying molecular mechanisms for the anti-TC effects of HHT, tandem mass tags-labeled proteomics analysis was conducted in TPC-1 cells treated with or without HHT (20 nM) for 48 h. A total of 787 differentially expressed proteins were identified according to fold change >1.5 and *p* value <0.05 criteria, among which 659 were downregulated and 128 were upregulated (Figures 2A and 2B; Table S1). The Kyoto Encyclopedia of Genes and Genomes (KEGG) analysis demonstrated that these differentially expressed proteins were significantly enriched for the PI3K/AKT signaling pathway (Figure 2C). Gene Ontology analysis revealed that HHT mainly affected the process related to response to peptides, collagen-containing extracellular matrix, and structural constituent of ribosome (Figures 2D–2F). The PI3K/AKT signaling pathway is one of the critical signaling pathways that participates in the initiation and progression of TC;^18^ therefore, this signaling pathway was selected for further investigation. To verify the relationship between the PI3K/AKT signaling pathway and HHT treatment, western blotting was conducted to detect the expression of some key proteins related to this signaling pathway. The results suggested that HHT decreased p-FAK, p-PI3K, and p-AKT expression levels dose dependently, uncovering that HHT indeed inactivated the PI3K/AKT signaling pathway in TC cells (Figures 2G and S5).

TIMP1 is a regulator of the initiation and progression of multiple cancers.^19^ It interacts with other proteins to cause aberrant regulation of downstream signaling pathways, among which the PI3K/AKT signaling pathway is one of the main TIMP1-related signaling pathways. A recent study demonstrated that TIMP1 promotes TC progression through activating the PI3K/AKT signaling pathway.^20^ Notably, as the proteomics revealed in the present study, TIMP1 was among the top 10 differentially expressed proteins after HHT treatment (Figures 2A and 2B). Based on these findings from previous studies and our current research, TIMP1 was selected as a protein of interest for further study. Western blotting analysis verified that HHT decreased TIMP1 protein expression levels dose dependently (Figures 2G and S5). Interestingly, the mRNA levels of TIMP1 were also declined dose dependently upon HHT treatment (Figure 2H). Considering these observations, we propose that the anti-TC effects of HHT may be associated with the downregulation of TIMP1 and inactivation of the FAK/PI3K/AKT signaling pathway.

### TIMP1 downregulation participates in the anti-TC effects of HHT

**Figure 3:**
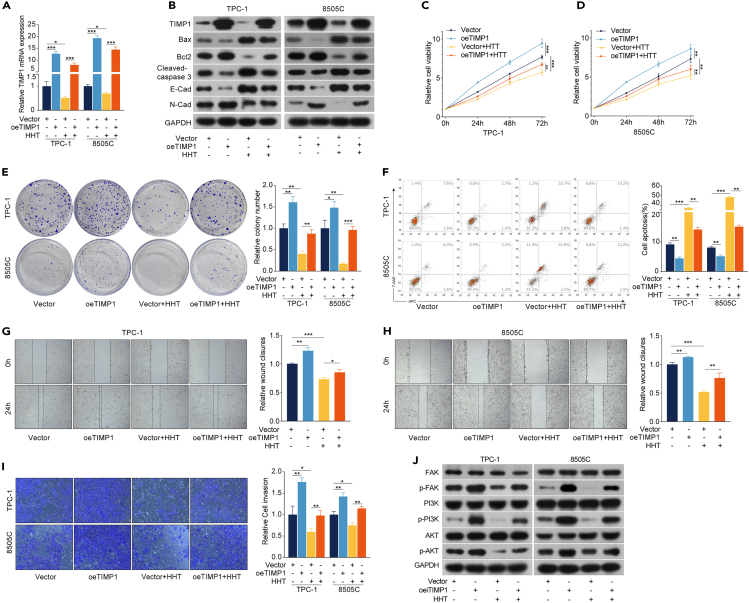
TIMP1 overexpression reserves the anti-TC effects of HHT (A) TPC-1 and 8505C cells were transfected with vector or TIMP1 overexpression (oeTIMP1) and then treated with HHT treatment for 48 h. RT-qPCR was performed to analyze the mRNA level of TIMP1. GAPDH was used as the control. (B) Western blotting analysis of the expression of TIMP1, Bax, Bcl2, cleaved caspase-3, E-cadherin, and N-cadherin in TPC-1/oeTIMP1 and 8505C/oeTIMP1 cells with HHT treatment for 48 h. GAPDH was used as the control. (C) and (D) CCK-8 assay was performed to detect cell viability of TPC-1/oeTIMP1 and 8505C/oeTIMP1 cells with HHT treatment for 24, 48, and 72 h. (E) Colony formation assay was used to detect the colony formation ability of TPC-1/oeTIMP1 and 8505C/oeTIMP1 cells with HHT treatment for 14 days. (F) Flow cytometry was performed to measure the effect of HHT on the apoptosis of TPC-1/oeTIMP1 and 8505C/oeTIMP1 cells with HHT treatment for 48 h. (G) and (H) Wound healing assay was performed to detect the cell migration rate. (I) Transwell assay was conducted to measure the cell invasion ability. (J) Western blotting analysis of the expression of FAK, p-FAK, PI3K, p-PI3K, AKT, and p-AKT in TPC-1/oeTIMP1 and 8505C/oeTIMP1 cells with HHT treatment for 48 h. GAPDH was used as the control. Data are presented as mean ± SD. ns, no significance, ∗*p* < 0.05, ∗∗*p* < 0.01, ∗∗∗*p* < 0.001.

**Figure 4:**
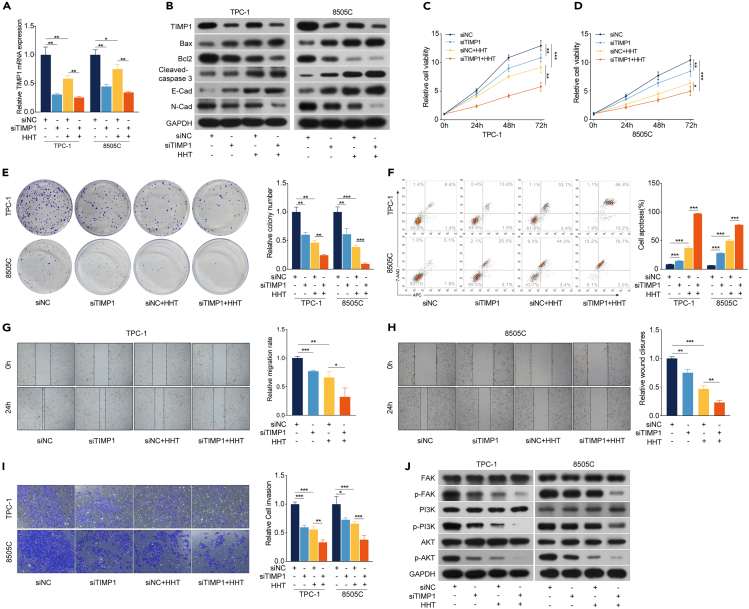
TIMP1 downregulation enhances the anti-TC effects of HHT (A) TPC-1 and 8505C cells were transfected with negative control (siNC) or TIMP1-targeting siRNA (siTIMP1) and then treated with HHT for 48 h. RT-qPCR was performed to analyze the mRNA level of TIMP1. GAPDH was used as the control. (B) Western blotting analysis of the expression of TIMP1, Bax, Bcl2, cleaved caspase-3, E-cadherin, and N-cadherin in TPC-1/siTIMP1 and 8505C/siTIMP1 with HHT treatment for 48 h. GAPDH was used as the control. (C) and (D) CCK-8 assay was performed to detect cell viability of TPC-1/siTIMP1 and 8505C/siTIMP1 cells with HHT treatment for 24, 48, and 72 h. (E) Colony formation assay was used to detect the colony formation ability of TPC-1/siTIMP1 and 8505C/siTIMP1 cells with HHT treatment for 14 days. (F) Flow cytometry was performed to measure the effect of HHT on the apoptosis of TPC-1/siTIMP1 and 8505C/siTIMP1 cells with HHT treatment for 48 h. (G) and (H) Wound healing assay was performed to detect the cell migration rate. (I) Transwell assay was conducted to measure the cell invasion ability. (J) Western blotting analysis of the expression of FAK, p-FAK, PI3K, p-PI3K, AKT, and p-AKT in TPC-1/siTIMP1 and 8505C/siTIMP1 with HHT treatment for 48 h. GAPDH was used as the control. Data are presented as mean ± SD. ns, no significance, ∗*p* < 0.05, ∗∗*p* < 0.01, ∗∗∗*p* < 0.001.

**Figure 5:**
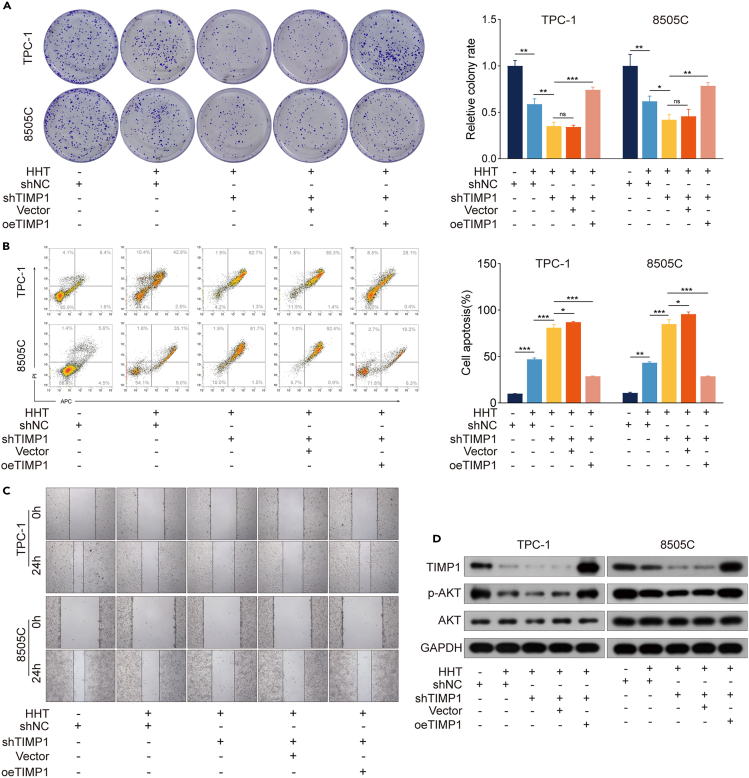
Re-expression of TIMP1 attenuates the enhancement of anti-TC effects of HHT induced by TIMP1 knockdown (A) TIMP1 expression in TPC-1 and 8505C cells was downregulated by transfecting with stable TIMP1 knockdown (shTIMP1) and re-expressed by transfecting with oeTIMP1. Colony formation assay was performed to detect the colony formation ability of TPC-1 and 8505C cells transfected with shTIMP1 and/or oeTIMP1 and treated with HHT for 14 days. (B) Wound healing assay was performed to detect the migration rate of TPC-1 and 8505C cells transfected with shTIMP1 and/or oeTIMP1 and treated with HHT. (C) Flow cytometry detected the effect of HHT on the apoptosis of TPC-1 and 8505C cells transfected with shTIMP1 and/or oeTIMP1 and treated with HHT for 48 h. (D) Western blotting analysis of the expression of TIMP1, AKT, and p-AKT in TPC-1 and 8505C cells transfected with shTIMP1 and/or oeTIMP1 and treated with HHT for 48 h. GAPDH was used as the control. Data are presented as mean ± SD. ns, no significance, ∗*p* < 0.05, ∗∗*p* < 0.01, ∗∗∗*p* < 0.001.

To verify the anti-TC effects of HHT are dependent on TIMP1 expression, TPC-1 and 8505C cells with TIMP1 overexpression (oeTIMP1) were constructed (Figures 3A, 3B, and S6A). oeTIMP1 potently promoted cell proliferation, invasion, and migration and inhibited apoptosis in TPC-1 and 8505C cells (Figures 3B–3I and S6A). Of note, the anti-tumor effects of HHT on TC cells were partially reversed by oeTIMP1 (Figures 3B–3I and S6A). Simultaneously, TPC-1 and 8505C cells were transiently transfected with TIMP1-targeting small interfering RNAs (siRNAs) (siTIMP1). The efficacy of transient transfection was detected and TIMP1 siRNA #1 was selected for subsequent experiments (Figures 4A, 4B, and S7). Contrary to oeTIMP1, siTIMP1 resulted in the inhibition of cell proliferation, invasion, and migration, as well as induction of apoptosis, in TPC-1 and 8505C cells (Figures 4B–4I and S8A). Besides, siTIMP1 enhanced the ability of HHT to exert anti-TC functions in TPC-1 and 8505C cells (Figures 4B–4I and S8A). To rigorously prove that TIMP1 indeed participates in the anti-TC effects of HHT, a TIMP1 knockdown and rescue experiment was performed. TIMP1 expression of TPC-1 and 8505C cells was downregulated by transfecting stable TIMP1 knockdown (shTIMP1) and then re-expressed by oeTIMP1. The colony formation assay, flow cytometry analysis, and wound healing assay revealed that re-expression of TIMP1 attenuated the enhancement of anti-TC effects of HHT induced by TIMP1 deletion, as evidenced by rescue of the proliferation and migration ability of TC cells (Figures 5A–5D and S9). These observations further support the notion that TIMP1 downregulation participates in the anti-TC effects of HHT.

### HHT restrains TC growth *in vivo*

**Figure 6:**
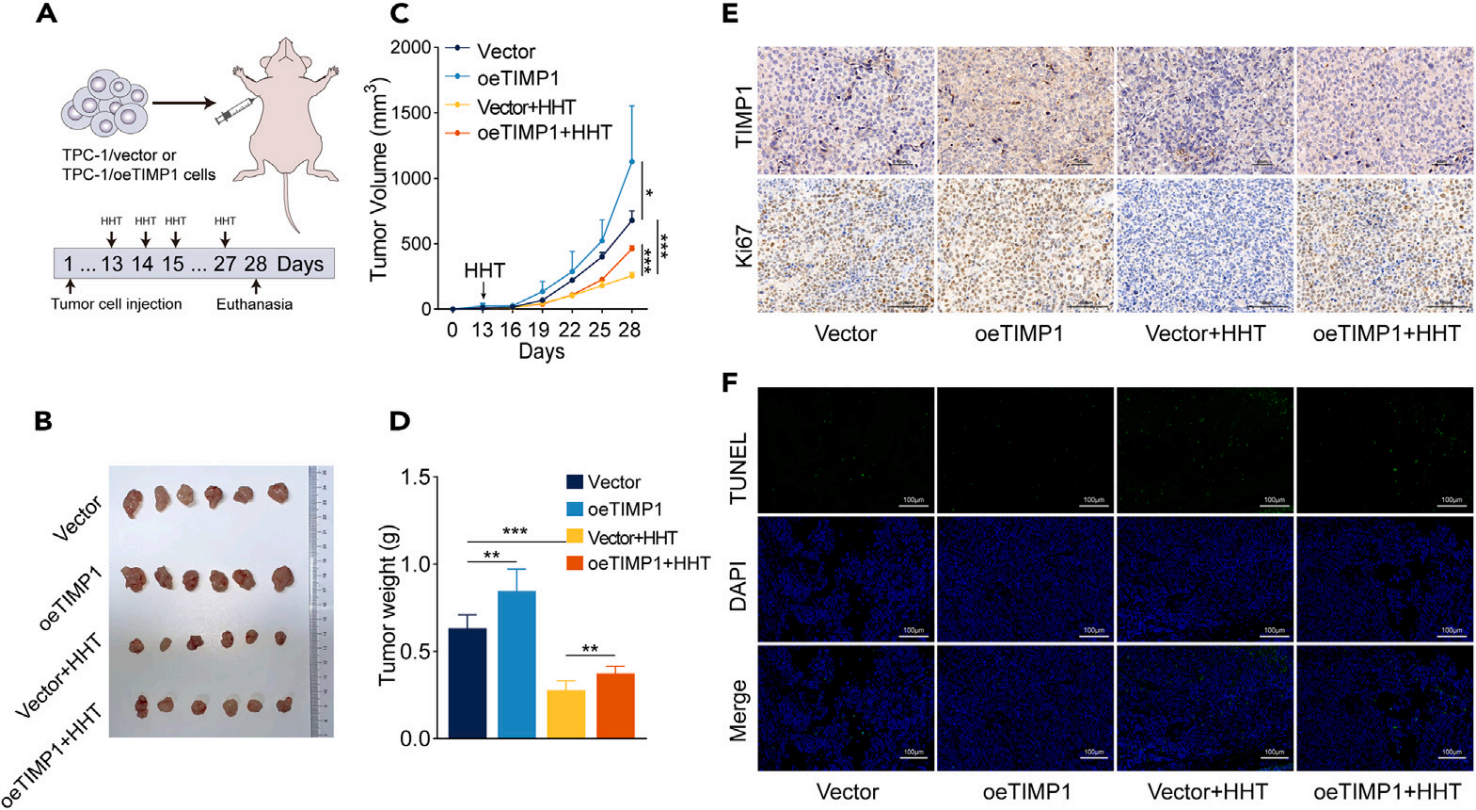
HHT restrains TC growth *in vivo* (A) The establishment of xenografts tumor model. (B) Images of tumor xenografts obtained at the end of the animal experiment. (C) The change of the tumor volume throughout the study. (D) The tumor weight at the end of the experiment. (E) Representative IHC staining images of TIMP1 and Ki-67 in xenograft tumors. (F) Representative TUNEL staining images of tumor tissues. Data are presented as mean ± SD. ∗*p* < 0.05, ∗∗*p* < 0.01, ∗∗∗*p* < 0.001.

To validate the anti-TC effects of HHT and the underlying mechanisms *in vivo*, subcutaneous xenograft tumor models were established in nude mice by injecting TPC-1/Vector or TPC-1/oeTIMP1 cells. Following a 12-day period of tumor establishment, HHT was used to treat tumor-bearing mice every day for an additional 15 days (Figure 6A). Consistent with *in vitro* findings, HHT treatment markedly decreased tumor burden *in vivo*, as evidenced by reduced tumor volume and tumor weight (Figures 6B–6D). Subsequently, immunohistochemistry (IHC) staining was performed on the xenograft tumor tissues and revealed that TIMP1 and Ki67 were downregulated in the HHT treatment group (Figure 6E). Additionally, the TUNEL assay revealed that the apoptotic cell proportion of tumor tissues was increased after HHT treatment (Figure 6F). These results demonstrate that HHT treatment inhibits TC tumor growth *in vivo*. Notably, oeTIMP1 promoted the growth of the xenograft tumor and reversed the anti-TC effects of HHT *in vivo* (Figures 6B–6F). Taken together, these results indicate that HHT treatment substantially inhibits TC growth *in vivo* by downregulating TIMP1.

### The FAK/PI3K/AKT signaling pathway is required for TC progression induced by TIMP1

The FAK/PI3K/AKT signaling pathway is reported as a main downstream of TIMP1 and is involved in the pro-tumor effects of TIMP1 in many cancers.^21^ We have found that this signaling pathway was inactive upon HHT treatment (Figures 2G and S5). Therefore, we proposed that HHT could inhibit the FAK/PI3K/AKT signaling pathway through downregulating TIMP1 expression in TC. Western blotting analysis suggested that oeTIMP1 markedly increased the levels of p-FAK, p-PI3K, and p-AKT and reversed the inhibitory effect of HHT on these proteins, while siTIMP1 decreased the levels of p-FAK, p-PI3K, and p-AKT and enhanced the inhibitory effects of HHT (Figures 3J, 4J, S6B, and S8B). Moreover, in the TIMP1 knockdown and rescue experiment, shTIMP1 markedly enhanced the inhibitory effects of HHT on p-AKT expression, while TIMP1 re-expression attenuated this enhancement phenomenon (Figures 5D and S9). These results suggest that HHT results in the inactivation of the FAK/PI3K/AKT signaling pathway by downregulating TIMP1 expression.

**Figure 7:**
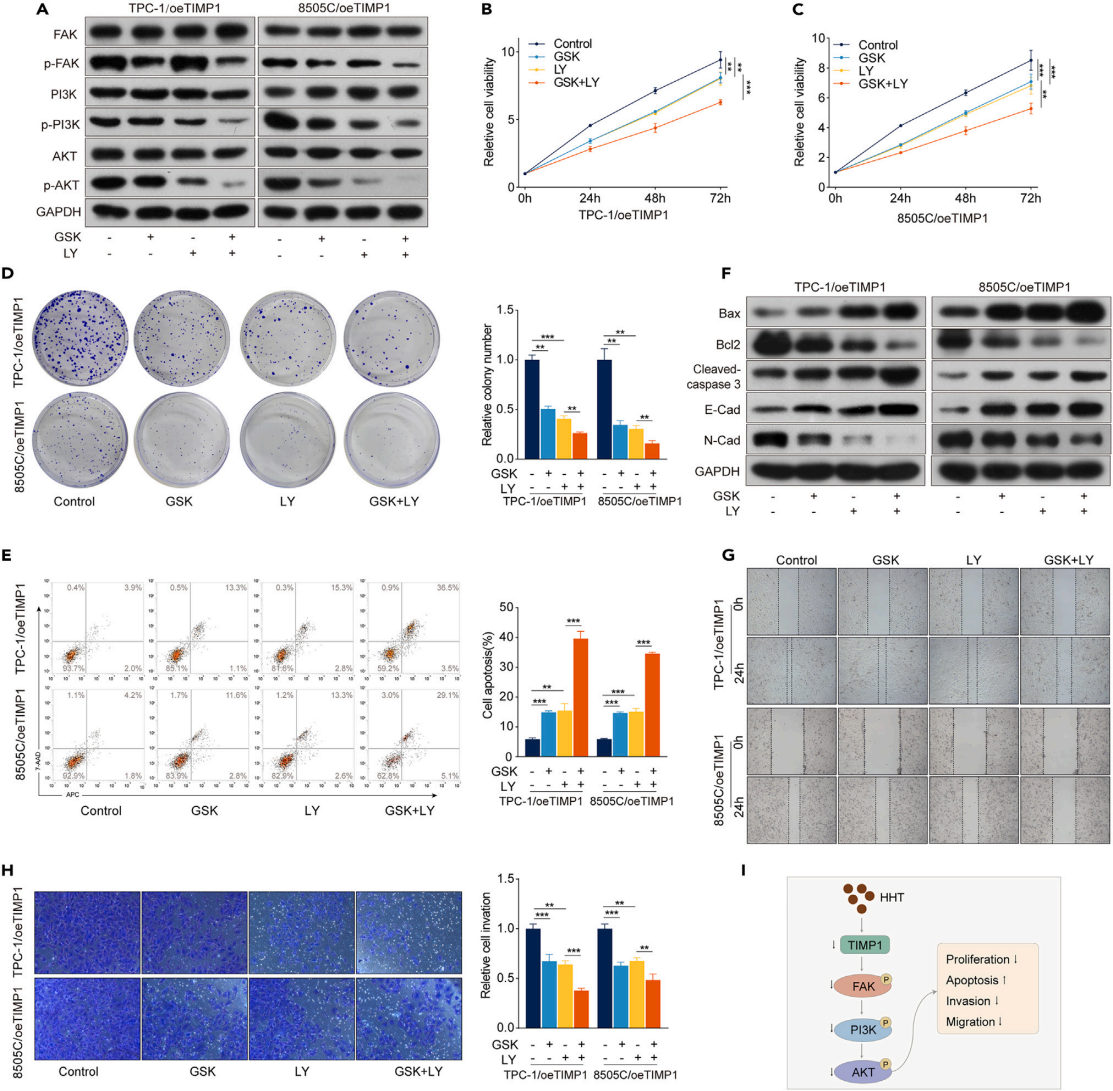
The FAK/PI3K/AKT signaling pathway is required for TC progression induced by TIMP1 (A) Western blotting analysis of the expression of FAK, p-FAK, PI3K, p-PI3K, AKT, and p-AKT in TPC-1/oeTIMP1 and 8505C/oeTIMP1 cells treated with GSK2256098 (GSK) and/or LY294002 (LY) for 48 h. (B) and (C) CCK-8 assay was performed to detect cell viability of TPC-1/oeTIMP1 and 8505C/oeTIMP1 cells treated with GSK and/or LY for 24, 48, and 72 h. (D) Colony formation assay was conducted to determine the clonogenic ability of TPC-1/oeTIMP1 and 8505C/oeTIMP1 cells treated with GSK and/or LY for 14 days. (E) Flow cytometry was used to detect the apoptosis of TPC-1/oeTIMP1 and 8505C/oeTIMP1 cells treated with GSK and/or LY for 48 h. (F) Western blotting analysis of the expression of Bax, Bcl2, cleaved caspase-3, E-cadherin, and N-cadherin in TPC-1/oeTIMP1 and 8505C/oeTIMP1 cells treated with GSK and/or LY for 48 h. GAPDH was used as the control. (G) Wound healing assay was performed to detect cell migration ability. (H) Transwell assay was performed to detect cell invasion ability. (I) HHT exerts anti-TC effects by inhibiting the TIMP1/FAK/PI3K/AKT signaling pathway. Data are presented as mean ± SD. ns, no significance, ∗*p* < 0.05, ∗∗*p* < 0.01, ∗∗∗*p* < 0.001.

To determine whether activation of the FAK/PI3K/AKT signaling pathway is required for TC progression caused by oeTIMP1, FAK inhibitor (GSK2256098) and PI3K inhibitor (LY294002) were employed to block this pathway. Western blotting analysis suggested that the FAK/PI3K/AKT signaling pathway was successfully inactivated, as evidenced by decreased levels of p-FAK, p-PI3K, and p-AKT after GSK2256098 treatment and decreased levels of p-PI3K and p-AKT after LY294002 treatment (Figures 7A and S10A). Subsequent biological experiments demonstrated that GSK2256098 or LY294002 treatment attenuated cell proliferation, invasion, and migration while induced apoptosis in TPC-1/oeTIMP1 and 8505C/oeTIMP1 cells (Figures 7B–7H and S10B). These data indicated that GSK2256098 or LY294002 treatment abolished tumor progression induced by oeTIMP1 in TC. Moreover, LY294002 enhanced the anti-tumor functions of GSK2256098 in TPC-1/oeTIMP1 and 8505C/oeTIMP1 cells (Figures 7B–7H and S10B). Collectively, these findings confirm the critical role of the FAK/PI3K/AKT signaling pathway in TC progression induced by TIMP1. Therefore, we propose that the HHT-induced anti-TC effects are mediated by inactivating the TIMP1/FAK/PI3K/AKT signaling pathway (Figure 7I).

### TIMP1 expression is upregulated in TC and associated with invasive clinical features

**Figure 8:**
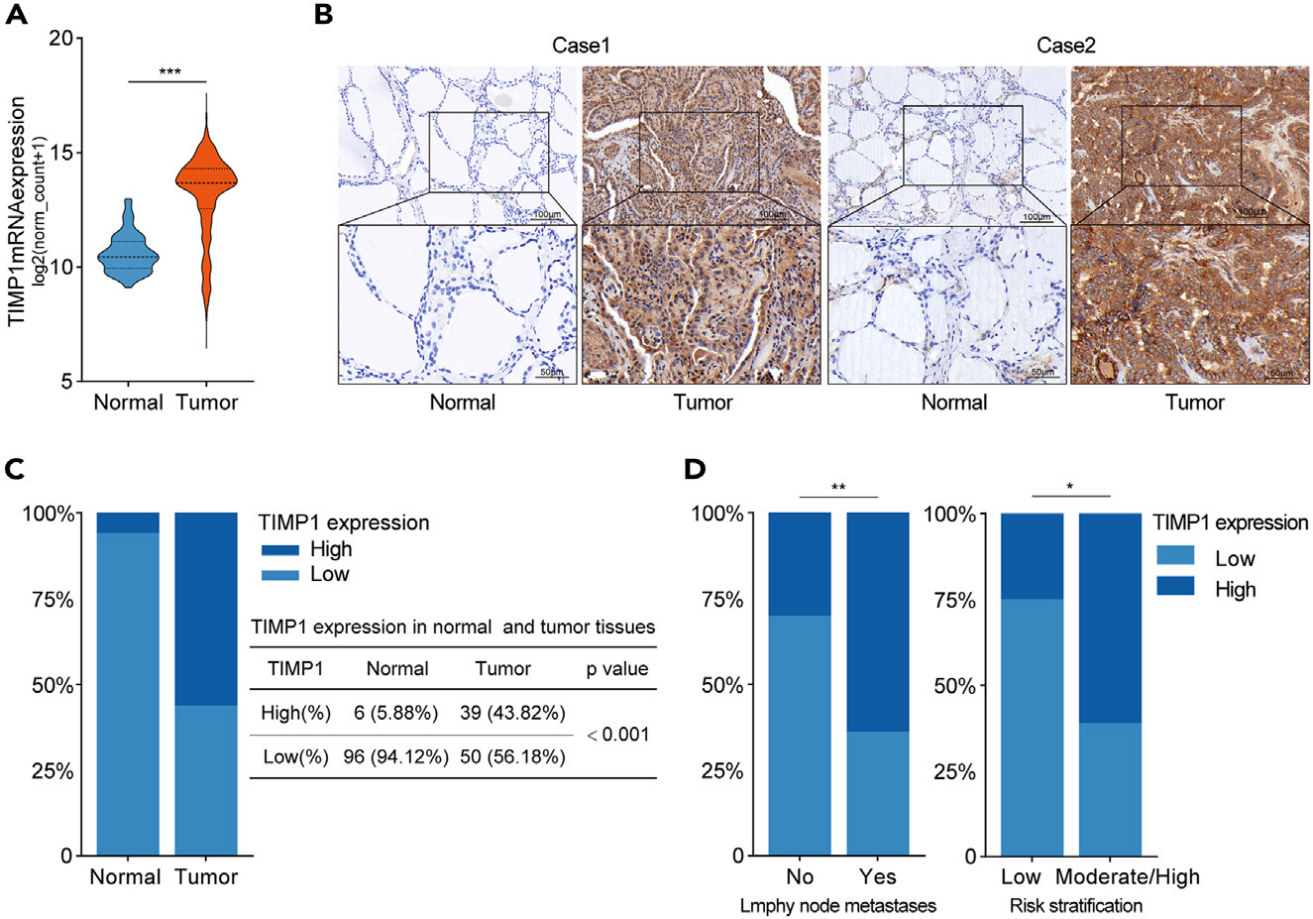
TIMP1 expression is upregulated in TC and associated with invasive clinical features (A) The mRNA expression level of TIMP1 in normal thyroid tissues and TC tissues from the TCGA database. (B) Representative IHC staining images of TIMP1 in TC and normal thyroid tissues. (C) TIMP1 expression in TC was higher than that in normal thyroid tissues based on immunoreactivity score. (D) High TIMP1 expression was correlated to lymph node metastases and higher risk stratification. ns, no significance; ∗*p* < 0.05, ∗∗*p* < 0.01, ∗∗∗*p* < 0.001.

To further verify the expression pattern of TIMP1 in TC, the mRNA levels of TIMP1 in TC tissues were analyzed using The Cancer Genome Atlas (TCGA) data. The result suggested that TIMP1 expression was significantly higher in TC tissues compared with normal thyroid tissues (Figure 8A). Then, IHC staining was performed to investigate the protein expression of TIMP1 in TC (*n* = 89) and normal thyroid tissues (*n* = 102). Representative IHC images are shown in Figure 8B. The samples with TIMP1-high expression accounted for 43.82% (39/89) in TC tissue samples while only 5.88% (6/102) in normal tissue samples (Figures 8C and S11), suggesting TIMP1 was significantly upregulated in TC tissues. To determine whether TIMP1 is clinically correlated to TC progression, we evaluated the association between TIMP1 expression and clinical features based on the IHC staining results. The detailed clinical information of patients is listed in Table S2. TIMP1-high expression significantly correlated to invasive clinical features, including lymph node metastases and higher risk stratification (Figure 8D; Table 1). Besides, the TCGA data analysis showed that TIMP1-high expression was correlated to invasive clinical features including extrathyroidal invasion, multifocality, lymph node metastases, higher T stage, and higher TNM stage (Figure S12). In brief, these results suggest that TIMP1 is upregulated in TC and could be a promising biomarker for invasive clinical features.

**Table 1:** The correlation between TIMP1 expression and clinical features in TC

	TIMP1 expression		Total	*p* value
	Low	High		
**Age (years)**				

<55	31	39	70	0.865
≥55	8	11	19	
Sex				0.053
Male	16	11	27	
Female	23	39	62	

**Pathology type**				

PTC	34	45	79	0.676
FTC	5	5	10	

**Extrathyroidal invasion**				

No	31	32	63	0.111
Yes	8	18	26	

**Multifocality**				

No	17	20	37	0.829
Yes	22	30	52	

**Tumor size (cm)**				

<2	27	33	60	0.747
≥2	12	17	29	

**Lymph node metastases**				

No	14	6	20	0.007^a^
Yes	25	44	69	

**Distant metastases**				

No	48	45	83	0.165
Yes	1	5	6	

**TNM stage**				

Ⅰ	31	39	70	0.865
Ⅱ/Ⅲ/Ⅳ	8	11	19	

**Risk stratification**				

Low	9	3	12	0.019^a^
Moderate/High	30	47	77	

a *p*＜0.05

## Discussion

Although most TC patients achieve effective responses and good prognoses with conventional therapies, some still suffer from aggressive diseases with poor prognoses. Encouraging breakthroughs of tyrosine kinase inhibitors-based treatments have been achieved in TC treatment in recent years; however, some patients show treatment failure due to drug resistance and untolerability.^22^ Therefore, there is still an urgent need to investigate novel and effective therapeutic strategies for TC treatment. Repurposing FDA-approved drugs for new indications could be a feasible approach to overcome current therapeutic obstacles and accelerate drug development in cancer treatment.^6^ Among FDA-approved drugs, natural compounds have recently received increasing attention for treating various cancers.^23^ HHT, an alkaloid derived from *Cephalotaxus*, has been approved to treat chronic myeloid leukemia for many years and was recently confirmed to have promising anti-tumor activity in numerous solid tumors.^8^^,^^9^^,^^10^^,^^11^ However, to our knowledge, the potential therapeutic functions of HHT in TC and the underlying mechanisms have not been elucidated so far. Herein, we prove that HHT impressively suppresses TC progression *in vitro* and *in vivo*, involving suppression of cell proliferation, invasion, and migration, as well as augmentation of apoptosis. Mechanistically, the HHT-induced anti-TC effects are mediated by the inactivation of the TIMP1/FAK/PI3K/AKT signaling pathway. Therefore, HHT could be an effective anti-TC agent and be repurposed for TC treatments in the future.

HHT restrains tumor progression by inhibiting some oncoproteins in various malignancies, such as EphB4 in hepatocellular carcinoma,^11^ PHGDH in neuroblastoma,^24^ and NF-κB in acute myelogenous leukemia.^25^ In the present study, proteomics and western blotting analysis suggested that TIMP1 is a functional protein downregulated by HHT in TC. Therefore, we speculate that TIMP1 is an oncoprotein participating in the anti-TC effects of HHT. TIMP1 belongs to the tissue inhibitor of the metalloproteinases family of proteins and exerts anti-proteolytic activity on matrix metalloproteinases (MMPs), which is initially proposed to prevent tumor progression by inhibiting MMP activity.^19^ Unexpectedly, these findings are inconsistent with the observations that aberrant upregulation of TIMP1 correlated to malignant progression and poor prognoses in numerous cancers, such as breast, liver, and lung cancer.^26^^,^^27^^,^^28^ Inspired by these paradoxical findings, some studies confirmed that TIMP1, independent of its inhibitory effect on MMPs, activates intercellular pro-survival signaling through binding membrane CD63 to accelerate tumorigenic biological processes.^19^ In TC, TIMP1 expression was reported to be higher in malignant thyroid nodules than in normal thyroid tissues and benign thyroid nodules.^29^^,^^30^ Consistently, we demonstrated again that TIMP1 was overexpressed in TC compared to normal thyroid tissues. We also confirmed that TIMP1 is an indicator of aggressive behavior in TC, evidenced by the strong correlation between high TIMP1 expression and invasive clinical features. This finding is in agreement with previous studies suggesting that TIMP1 is upregulated in TC tissues with cervical lymph node metastasis^31^ and locally invasive PTC tissues.^32^ Besides, TIMP1 is reportedly as a tumor-secreted protein that could be detected in the serum.^28^ Several studies found that the serum levels of TIMP1 are higher in the patients with DTC compared to the patients with benign nodule.^33^^,^^34^ However, we did not evaluate the serum levels of TIMP1 of TC patients in the present study. Growing evidence suggests TIMP1 impacts on various cellular processes and regulates the initiation and progression in a wide range of cancers.^35^^,^^36^ Our experiments showed that TIMP1 facilitated cell proliferation, invasion, and migration and inhibited apoptosis of TC *in vitro* and *in vivo*. Of note, exogenous TIMP1 expression attenuated the anti-TC effects of HHT while deletion of TIMP1 contributed to a contrary effect. Moreover, re-expression of TIMP1 attenuated the enhancement of the anti-TC effects of HHT induced by TIMP1 knockdown. These results support the notion that HHT exerts its anti-TC activity by downregulating TIMP1 expression.

Intriguingly, we found that HHT decreased TIMP1 expression at the mRNA level, suggesting that the HHT-induced downregulation of TIMP1 protein expression was at least in part due to the inhibition of TIMP1 transcription. This speculation is in line with previous studies that revealed HHT regulates the transcription of several oncogenes. For example, HHT robustly inhibits INSM1 promotor activity resulting in cell apoptosis in neuroblastoma^37^ and blocks nuclear translocation of p-STAT3 and its transcriptional activities on multiple genes in pancreatic cancer.^38^ Nevertheless, as a protein synthesis inhibitor, HHT prevents aminoacyl-tRNA binding to ribosomes, resulting in the inhibition of ribosomes from performing protein synthesis.^39^ Besides, some studies reported that HHT directly binds to some oncoproteins and inhibits their expression to exert anti-tumor effects, such as PHGDH in neuroblastoma^24^ and NF-κB in acute myelogenous leukemia.^25^ The mechanisms for the inhibitory effects of HHT on TIMP1 expression, which are likely to be multifactorial, are not been fully understood. Results of the present study only preliminarily suggest that HHT inhibits TIMP1 transcription, and it remains a question whether HHT downregulates TIMP1 expression through its canonical protein synthesis inhibition manner. Further studies are needed to uncover the potential mechanisms of HHT on the regulation of TIMP1 expression, especially its canonical protein synthesis inhibition activity and whether it directly binds to TIMP1 protein.

Previous studies reported that HHT exerts anti-tumor effects by regulating several signaling pathways, including the MEK/ERK, PI3K/AKT, and JAK2/STAT3 signaling pathway.^8^^,^^9^^,^^38^^,^^40^ To find the relevant signaling pathway for HHT in TC, KEGG analysis was conducted based on proteomics data, which demonstrated that HHT mainly regulates the PI3K/AKT signaling pathway. Abnormal activation of the PI3K/AKT signaling pathway affects numerous fundamental cellular processes, including cell proliferation, apoptosis, and differentiation.^41^ Considering that the PI3K/AKT signaling is a crucial pro-tumor pathway that is involved in the numerous cellular biological processes in TC,^18^ we further confirmed that the FAK/PI3K/AKT signaling pathway is critical for the anti-TC effects of HHT. This was consistent with previous studies reporting that the PI3K/AKT signaling pathway participates in the inhibiting activity of HHT in colorectal cancer^40^ and hepatocellular carcinoma.^42^ TIMP1 interacts with other proteins to cause aberrant regulation of downstream signaling pathways, among which the PI3K/AKT pathway is one of the main TIMP1-related signaling pathways.^19^ In colon cancer, TIMP1 promotes cell proliferation and metastasis by activating the FAK/PI3K/AKT pathway, and selective FAK or AKT inhibitors impair the pro-tumor effects of TIMP1.^43^^,^^44^ To confirm that the activation of the FAK/PI3K/AKT signaling pathway is required for TIMP1-induced tumor progression in TC, we performed biological experiments and found that the FAK/PI3K/AKT signaling pathway was activated by exogenous TIMP1 expression or inhibited by deletion of TIMP1 expression, and selective FAK or PI3K inhibitors abolish the stimulation of TIMP1 on the progression of TC. These findings are consistent with a recent report that TIMP1 forms a complex with CD44 variant isoform 6 and thus sustains TC growth through activating the PI3K/AKT signaling pathway.^20^ Therefore, we propose that the anti-TC effects of HHT are mediated by the inactivation of the TIMP1/FAK/PI3K/AKT signaling pathway.

Different from the selective targeting mechanism of TKIs, HHT is a broad-spectrum protein inhibitor that does not target specific proteins. The underlying regulatory mechanisms of anti-tumor effects carried out by HHT are relatively complex that involve not only inhibition of signaling pathways, but also the regulation of other cellular processes including epigenetic modification,^8^ metabolic reprograming,^24^ and immune microenvironment.^45^ Notably, proteomics analysis in the present study showed that many oncogenic proteins, except for TIMP1, were affected by HHT, suggesting that the inhibitory effect of HHT on oncoproteins is not specific to TIMP1. Thus, the anti-TC effects of HHT cannot be only attributed to TIMP1 downregulation. Although we clarified that HHT could be an efficient inhibitor of TIMP1 in TC treatment, further studies to expand the validation of other proteins would provide more novel information to reveal the complex regulatory mechanisms for HHT from multiple perspectives.

In conclusion, our study demonstrates that the FDA-approved drug HHT exerts strong anti-TC effects by downregulating the TIMP1 expression and then inactivating the FAK/PI3KAKT signaling pathway. Our discoveries provide a possible therapeutic strategy for TC based on repurposing HHT.

### Limitations of the study

The present study has a few limitations. First, although we confirmed the anti-TC effects of HHT through a series of preclinical studies, whether HHT could be used in clinical practice needs further investigation. Besides, previous studies have suggested that the serum level of TIMP1 was upregulated in TC patients; however, only TC tissue samples were used for TIMP1 expression detection in our study, and the significance of TIMP1 expression in serum samples still needs to be studied. Moreover, we only confirmed that the TIMP1/FAK/PI3K/AKT signaling pathway participates in the anti-TC effects of HHT; additional studies should be performed to verify the role of other proteins to fully elucidate the multifarious mechanisms of HHT.

## STAR★Methods

### Key resources table

**Table:** 

REAGENT or RESOURCE	SOURCE	IDENTIFIER
**Antibodies**		

Rabbit polyclonal anti-Bax	Proteintech	Cat# 50599-2-lg; RRID: AB_2061561
Rabbit polyclonal anti-Bcl2	Millipore	Cat#ab1722; RRID: AB_569393
Rabbit monoclonal anti-Cleaved caspase-3	Cell Signaling Technology	Cat# 9664T; RRID: AB_2070042
Rabbit polyclonal anti-E-cadherin	Proteintech	Cat# 20874-1-AP; RRID: AB_10697811
Rabbit polyclonal anti-N-cadherin	Proteintech	Cat# 22018-1-AP; RRID: AB_2813891
Rabbit polyclonal anti-NIS	Abcam	Cat# ab83816; RRID: AB_1860904
Rabbit monoclonal anti-TIMP1	Abcam	Cat# ab211926
Rabbit polyclonal anti-FAK	Cell Signaling Technology	Cat# 3285; RRID: AB_2269034
Rabbit monoclonal anti-phospho-FAK	Cell Signaling Technology	Cat# 8556s; RRID: AB_10891442
Rabbit monoclonal anti-PI3K	Zen bio	Cat#R22768
Rabbit polyclonal anti-Phospho-PI3K	Cell Signaling Technology	Cat# 4228; RRID: AB_659940
Rabbit monoclonal anti-AKT	Cell Signaling Technology	Cat# 4691; RRID: AB_915783
Rabbit monoclonal anti-Phospho-AKT	Cell Signaling Technology	Cat# 4060S; RRID: AB_2315049
Rabbit polyclonal anti-Ki-67	Abcam	Cat# ab15580; RRID: AB_443209
Rabbit monoclonal anti-GAPDH	Cell Signaling Technology	Cat# 5174; RRID: AB_10622025

**Bacterial and virus strains**		

Lentivirus	Asia Vector Biotechnology (Shanghai, China)	N/A

**Biological samples**		

Human thyroid cancer tissue	Shanghai Sixth People’s Hospital	N/A

**Chemicals, peptides, and recombinant proteins**		

Homoharringtonine	DC Chemicals (Shanghai, China)	Cat# DC9650
GSK2256098 (FAK inhibitor)	Selleck	Cat# S8523
LY294002 (PI3K inhibitor)	Selleck	Cat# S1105
Lipofectamine 2000	Invitrogen	Cat# 11668030
TRIzol reagent	Invitrogen	Cat# 15596026

**Critical commercial assays**		

CCK-8 assay	BBI Life Sciences (Shanghai, China)	Cat# E606335-0500
Annexin V-APC/7-AAD Apoptosis Kit	Elabscience (Wuhan, China)	Cat# E-CK-A218
Annexin V-APC/PI Apoptosis Kit	Elabscience (Wuhan, China)	Cat# E-CK-A217
TransScript All-in-One First-Strand cDNA Synthesis SuperMix	TransGen (Beijing, China)	Cat# AT341
AceQ Universal SYBR qPCR Master Mix	Vazyme (Nanjing, China)	Cat# Q511-03
One Step TUNEL Apoptosis Assay Kit	Beyotime (Shanghai, China)	Cat# C1086

**Deposited data**		

Mass spectrometry proteomics data	iProx Consortium	PXD050295
TCGA-THCA	Open-source	https://xena.ucsc.edu

**Experimental models: Cell lines**		

Human: TPC-1	Chinese Academy of Sciences	N/A
Human: BCPAP	Chinese Academy of Sciences	N/A
Human: FTC-133	Chinese Academy of Sciences	N/A
Human: 8505C	Chinese Academy of Sciences	N/A

**Experimental models: Organisms/strains**		

Mouse: BALB/c nude	Jihui Laboratory Animal (Shanghai, China)	N/A

**Oligonucleotides**		

siRNA targeting sequence for TIMP1, see Table S3	RiboBio (Guangzhou, China)	N/A
shRNA targeting sequence for TIMP1, see Table S3	Asia Vector Biotechnology (Shanghai, China)	N/A
Primers for TIMP1, NIS, and GAPDH, see Table S4	This paper	N/A

**Recombinant DNA**		

pcDNA3.1-3×Flag-TIMP1	Asia Vector Biotechnology (Shanghai, China)	N/A

**Software and algorithms**		

R version 4.2.3	R Development Core Team	https://www.r-project.org
SPSS Statistics version 23.0	IBM	https://www.ibm.com/cn-zh/products/spss-statistics
GraphPad Prism version 9.0	GraphPad Software	https://www.graphpad.com

### Resource availability

#### Lead contact

Further information and requests for resources and reagents should be directed to and will be fulfilled by the lead contact, Zhonling Qiu (qiuzhongling123@163.com).

#### Materials availability

This study did not generate new unique reagents.

#### Data and code availability

• The proteomics data of TPC-1 cells treated with HHT have been deposited at the ProteomeXchange Consortium (https://www.iprox.cn/) and are publicly available as of the date of publication. Accession numbers are listed in the key resources table.
• This paper does not report original code.
• Any additional information required to reanalyze the data reported in this paper is available from the lead contact upon request.

### Experimental model and study participant details

#### Human subject

The thyroid cancer tissue microarray consisting of 89 tumor tissues and 102 adjacent normal tissues was established based on the surgical specimens obtained from patients with DTC between 2014 and 2017. The use of human subjects was approved by the Clinical Research Ethics Committee of Shanghai Sixth People’s Hospital Affiliated to Shanghai Jiao Tong University School of Medicine and was performed following the Declaration of Helsinki. Written informed consents were obtained from all patients. The detailed information of cinical characteristics is listed in Table S2.

#### Animals

Male BALB/c nude mice (4-6week) were purchased from Jihui Laboratory Animal Company (Shanghai, China) and housed under pathogenn-free conditions. The animal experiments was approved by the Animal Ethics Committee of Shanghai Sixth People’s Hospital Affiliated to Shanghai Jiao Tong University School of Medicine.

#### Cell lines

The human TC cell lines (TPC-1, BCPAP, FTC-133, and 8505C) were purchased from the Cell Bank of Type Culture Collection of the Chinese Academy of Sciences (Shanghai, China). All cell lines were cultured in RPMI 1640 medium (Gibco, USA) supplemented with 10% fetal bovine serum (FBS, Gibco, USA) and 1% penicillin/streptomycin at 37°C and 5% CO_2_.

### Method details

#### Cell transfection

For transient TIMP1 knockdown, small interfering RNAs targeting TIMP1 (siTIMP1) were obtained from RiboBio (Guangzhou, China). TPC-1 and 8505C cells were transfected with siTIMP1 using Lipofectamine 2000 (Invitrogen, USA) according to the manufacturer’s instructions. For stable TIMP1 overexpression or knockdown, premade lentiviral particles for TIMP1 overexpression or empty vector (oeTIMP1/Vector) and TIMP1 knockdown or negative control (shTIMP1/shNC) were obtained from Asia Vector Biotechnology (Shanghai, China). TPC-1 and 8505C cells were transfected with the indicated lentiviral reagent and then selected by antibiotics treatment. The efficiency of TIMP1 overexpression or downregulation was confirmed by qPCR or western blotting. The siRNAs and shRNAs sequences are listed in Table S3.

#### Cell counting Kit-8 (CCK-8) assay

Cell viability was detected using CCK-8 assay (BBI Life Sciences, China). Between 5×10^3^ and 8×10^3^ cells were seeded in 96-well plates and subsequently treated with HHT. Sample absorbance values were measured using a microplate reader.

#### Colony formation assay

For colony formation assay, 1×10^3^ cells were seeded in 6-well plates and treated with or without HHT for 14 days. The viable colonies were fixed with 4% paraformaldehyde for 15 minutes and then stained with crystal violet. The colonies were photographed and counted using ImageJ software.

#### Flow cytometry apoptosis analyses

Cell apoptosis rates were analyzed using an Annexin V-APC/7-AAD Apoptosis Kit (Elabscience, China) or Annexin V-APC/PI Apoptosis Kit (Elabscience, China) according to the manufacturer’s instructions. Cells were incubated with HHT in 6-well plates and washed twice with cold PBS. Subsequently, cells were incubated with Annexin V-APC and 7-AAD or PI for 20 minutes at room temperature. The percentage of apoptotic cells was measured using Attune NXT flow cytometry (Thermo Fisher Scientific, USA).

#### Transwell assay assay

For the Transwell assay, 2×10^4^ cells were resuspended in FBS-free medium and seeded into Transwell chambers (8.0 μm pore size, Corning, USA) with Matrigel-precoated (Corning, USA). Then, medium with 10% FBS was added to the lower well. After incubation for 24 hours, cells were fixed with 4% paraformaldehyde, stained with crystal violet, and photographed.

#### Wound healing assay

For wound healing assay, 6×10^5^ cells were seeded in 6-well plates. A “wound” was created using a sterile tip when the cells reached full confluence in the culture plates. Subsequently, the cells were photographed using a microscope at the 0-hour and 24-hour timepoints.

#### RNA extraction and real-time quantitative PCR (RT-qPCR)

Total RNA was isolated from samples using TRIzol reagent (Invitrogen, USA) according to the manufacturer’s instructions. Subsequently, RNA was reverse transcribed into cDNA using the TransScript All-in-One First-Strand cDNA Synthesis SuperMix (TransGen, China). Then, RT-qPCR was conducted with AceQ Universal SYBR qPCR Master Mix (Vazyme, China). GAPDH was used as the control. Data analysis was conducted using the 2^−ΔΔCt^ method. The primers used in the present study are shown in Table S4.

#### Western blotting

Protein expression was detected by western blotting as previously described.^46^^,^^47^ RIPA lysis buffer with protease inhibitors was used for protein isolation. After being separated using SDS-PAGE, protein samples were then transferred to PVDF membranes. The membranes were blocked with 5% BSA for 1 hour at room temperature, incubated with primary antibodies overnight at 4°C, and incubated with secondary antibodies for 1 hour at room temperature. The antibodies used in this study are listed in Table S5.

#### Proteomics analysis

TPC-1 cells were treated with HHT (20 nM) for 48 hours and subsequently collected for protein extraction. The protein samples were digested into peptides using trypsin (Promega, USA) and subsequently desalted using a C18 column. The concentration of peptides was measured using Pierce™ Quantitative Colorimetric Peptide Assay (Thermo Fisher Scientific). Subsequently, the peptide mixtures were dissolved in buffer A (20 mM ammonium formate in water, pH 10.0) and separated by high pH separation using the Ultimate 3000 system (Thermo Fisher Scientific) with a reverse phase column XBridge C18 (2.1 mm × 150 mm, 3 μm, Waters Corporation, USA). High pH separation was performed using a linear gradient, starting from 5% to 45% buffer B (20 mM ammonium formate in 80% acetonitrile, pH 10.0) over 40 minutes. Thermo Scientific Orbitrap Fusion™ coupled with the EASY-nLC 1100 system was used to analyze peptide mixtures. Tandem mass spectra were analyzed using Proteome Discoverer (Thermo Fisher Scientific), and differentially expressed proteins were identified according to the criteria of *P* < 0.01 and fold change > 1.5. The KEGG and GO analyses were performed in R software.

#### Animal experiments

The establishment of a subcutaneous xenograft tumor model was referred to our previous study.^48^ TPC-1 cells (5×10^6^) expressing either empty vector or overexpressing TIMP1 were subcutaneously injected into the right armpits of nude mice. Following a two-week period of tumor establishment, tumor-bearing mice were treated with HHT (irrigation stomach, 1 mg/kg, every day) or PBS for an additional 14 days and divided into four groups as follows (six mice/group): Vector, oeTIMP1, Vector + HHT, and oeTIMP1 + HHT. Tumor size was measured every three days after tumor establishment for two weeks. Tumor volumes were calculated using the following formula: Volume (mm^3^) = (length×width^2^)/2. The tumor-bearing mice were euthanized on the 28th day. The tumor tissues were excised, weighed, photographed, and subjected to IHC staining and TUNEL staining.

#### TUNEL staining

TUNEL staining was conducted utilizing the One Step TUNEL Apoptosis Assay Kit (Beyotime, China) following the manufacturer’s instructions. Briefly, tissue slides were incubated with Triton X-100/0.1% sodium citrate solution for 2 minutes on ice. After washing, tissue slides were exposed to the TUNEL reaction mixture and incubated at room temperature for 1 hour. Finally, the tissue slides were photographed using a fluorescence microscope.

#### Immunohistochemistry (IHC)

IHC staining was conducted on a thyroid cancer tissue microarray established with formalin-fixed and paraffin-embedded surgical specimens.^48^ Primary antibodies against TIMP1 (ab211926, Abcam, UK) were used. The level of TIMP1 expression was evaluated using the immunoreactivity score (IRS) calculated by multiplying the positive cell proportion (0: 0%, 1: 1% to 25%, 2: 26% to 50%, 3: 51% to 75%, 4: 76% to 100%) and staining intensity (0: negative, 1: weak, 2: moderate, 3: strong).^49^ An IRS value of ≤ 6 was regarded as low expression, while IRS > 6 indicated high expression. For IHC staining in xenograft tumors, tumor tissues were sliced into 4.0 μm-thick sections. The tissue slides were deparaffinized, hydrated, and subjected to antigen retrieval. Then, the slides were incubated with primary antibodies against Ki-67 (ab15580, Abcam, UK) and TIMP1 (ab211926, Abcam, UK). After secondary antibody incubation and nuclear staining, the slides were imaged using a microscope.

#### Database analysis

The RNA-seq data of the TCGA-THCA cohort was acquired from the UCSC Xena database (https://xena.ucsc.edu/),^50^ which included 59 normal thyroid tissue and 505 TC tissue samples.

### Quantification and statistical analysis

SPSS 26.0 (IBM, USA) and GraphPad Prism 9.0 (GraphPad, USA) were used for statistical analysis. Data are shown as the mean ± standard deviation (SD). Differences between groups were estimated using the two-tailed Student’s t-test. The correlation between TIMP1 expression and clinical features was determined by the χ^2^ test or Fisher’s exact test. Statistical significance was defined as a *p*-value less than 0.05.

## Supplemental information

Document S1. Figures S1–S12 and Tables S3–S5

Table S1. The differentially expressed proteins identified by proteomics, related to Figure 2

Table S2. Clinical information of the patients, related to Table 1 or STAR Methods
