# Supplementary material for: Repurposing homoharringtonine for thyroid cancer treatment through TIMP1/FAK/PI3K/AKT signaling pathway
Source: iScience. 2024 Apr 26;27(6):109829. doi: 10.1016/j.isci.2024.109829 (PMC11103377; doi:10.1016/j.isci.2024.109829)
Supplement: Document S1. Figures S1–S12 and Tables S3–S5 [file mmc1.pdf]

iScience, Volume *27*

Supplemental information

Repurposing homoharringtonine

for thyroid cancer treatment

through TIMP1/FAK/PI3K/AKT signaling pathway

Chuang Xi, Guoqiang Zhang, Nan Sun, Mengyue Liu, Nianting Ju, Chentian Shen, Hongjun Song, Quanyong Luo, and Zhongling Qiu

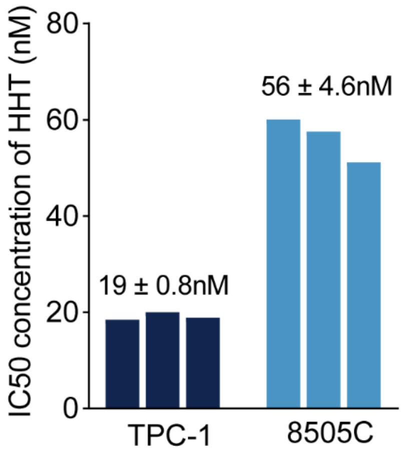

Figure S1. The IC50 concentration of HHT for TPC-1 and 8505C cells, related to Figure 1. The IC50 concentration of HHT for TPC-1 and 8505C cells was 19 ± 0.8nM and 56 ± 4.6 nM, respectively. Data are presented as mean ± SD.

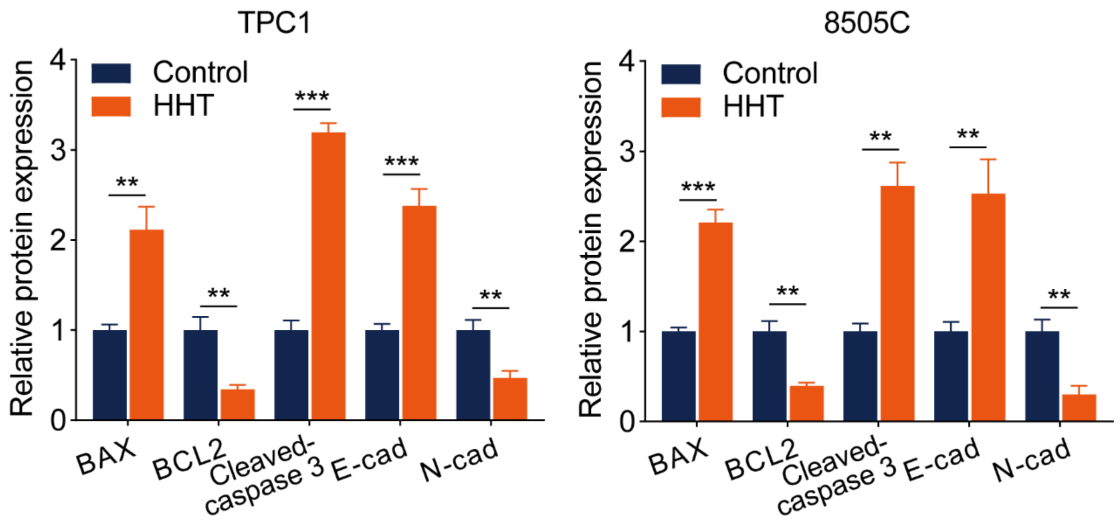

Figure S2. Semi-quantitative analysis of protein expression in Figure 1F, related to Figure 1. Semi-quantification analysis of apoptosis- and EMT-related proteins in TPC-1 and 8505C cells with HHT treatment for 48h (Fig. 1F). GAPDH was used as the control. Data are presented as mean ± SD. **$p < 0.01$, ***$p < 0.001$.

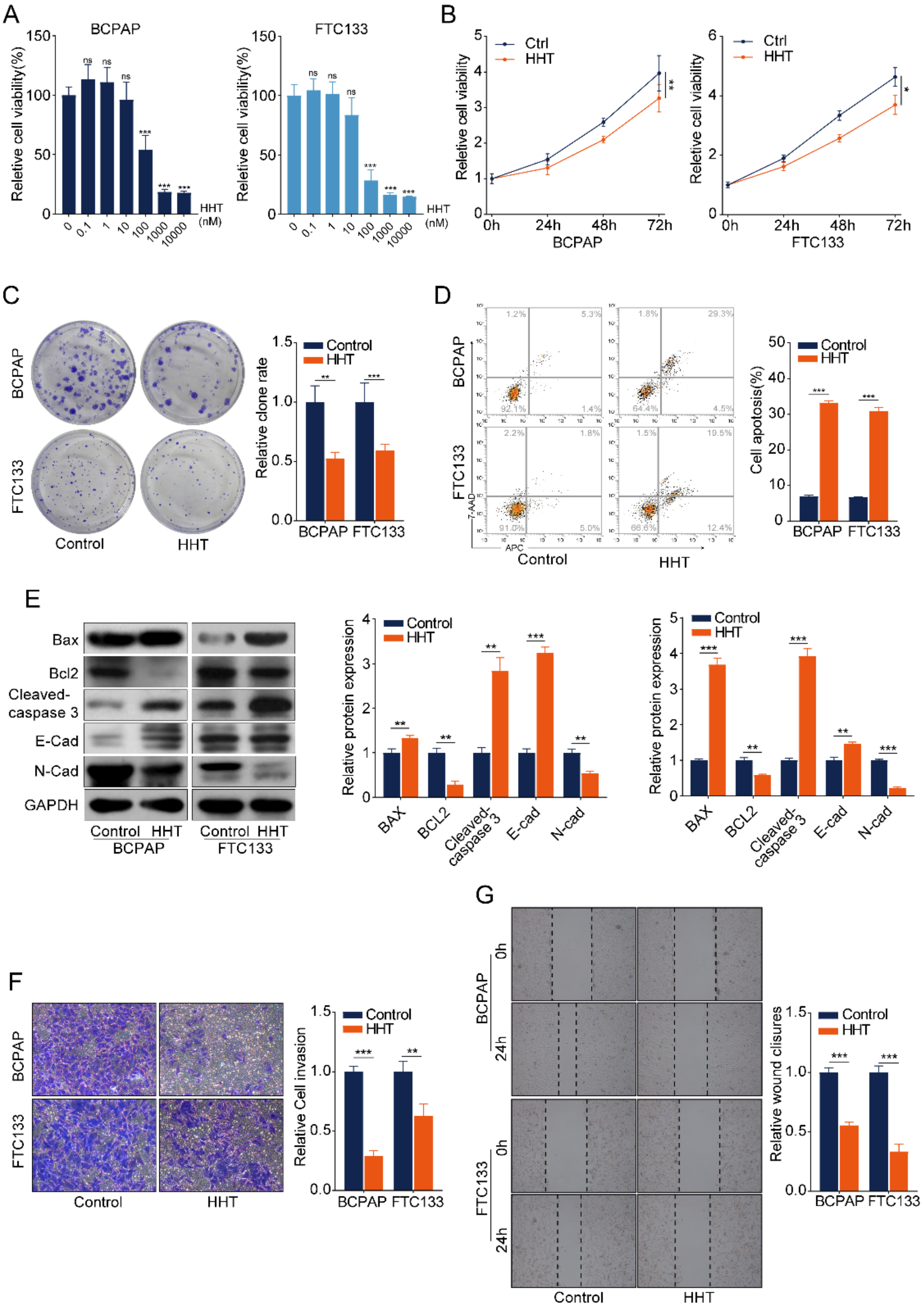

Figure S3. HHT suppresses BCPAP and FTC-133 cells *in vitro,* related to Figure 1. (A) CCK8 assay was performed to detect cell viability of BCPAP and FTC-133 cells treated with increased gradients of HHT for 48 h. **(B)** CCK8 assay was perfprmed to detect cell viability of BCPAP and FTC-133 cells with HHT treatment for 24, 48, and 72h. **(C)** Colony formation assay was used to detect the clonogenic ability of BCPAP and FTC-133 cells with HHT treatment for 14 days. **(D)** The effect of HHT on the cell apoptosis was detected by flow cytometry. **(E)** Western blotting analysis of the expression of Bax, Bcl2, Cleaved-caspase 3, E-cadherin, and N-cadherin with HHT treatment for 48h. GAPDH was used as the control. **(F)** Transwell assay was used to detect the cell invasion ability. **(G)** The wound healing assay was performed to detect the cell migration rate. Data are presented as mean ± SD. ns, no significance, $*p < 0.05$, $**p < 0.01$, $***p < 0.001$.

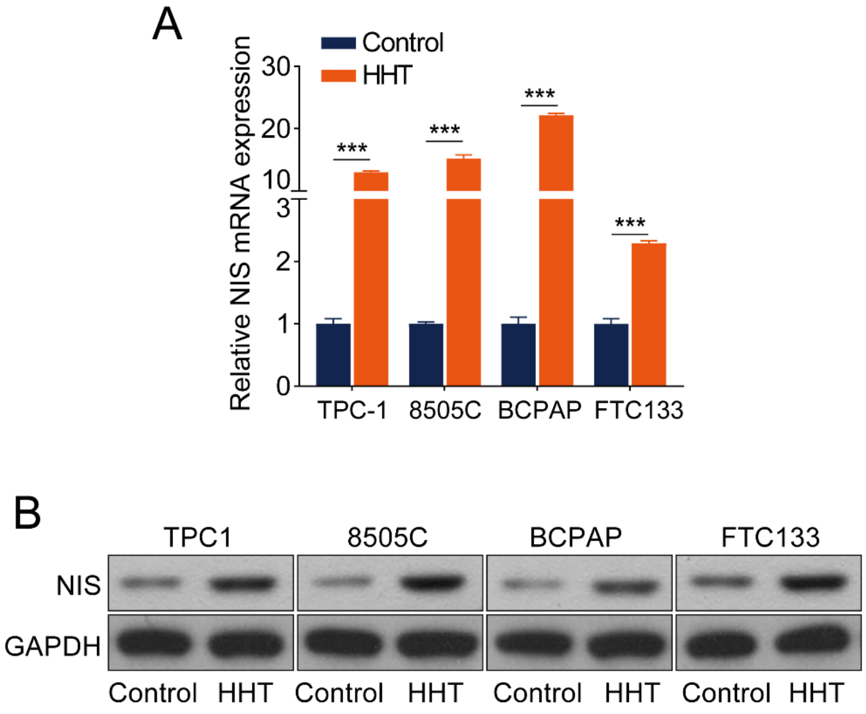

Figure S4. HHT downregulates the expression of NIS at both mRNA and protein levels, related to Figure 1. (A) RT-qPCR was performed to analyze the mRNA level of NIS in TC cells after HHT treatment for 48h. GAPDH was used as the control. **(B)** Western blotting analysis of the expression of NIS in TC cells with HHT treatment for 48h. GAPDH was used as the control. Data are presented as mean ± SD. ***p < 0.001.

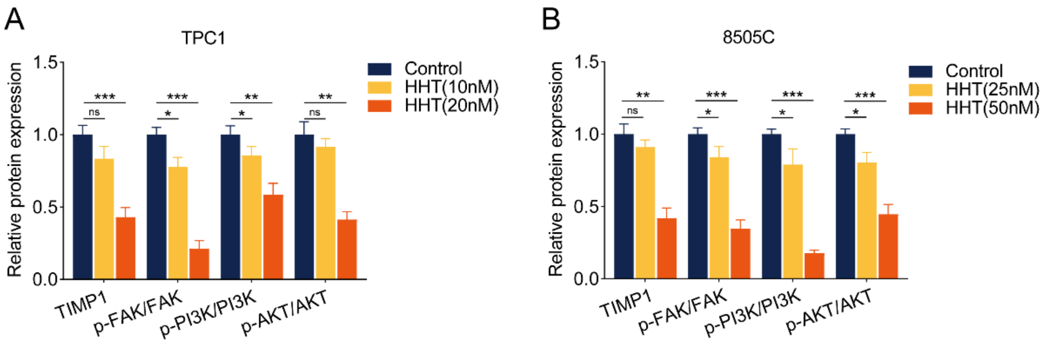

Figure S5. Semi-quantitative analysis of protein expression in Figure 2G, related to Figure 2. Semi-quantification analysis of TIMP1, FAK, PI3K, AKT, p-FAK, p-PI3K, and p-AKT expression in TPC-1 and 8505C cells with different concentrations of HHT treatment for 48h. GAPDH was used as the control. Data are presented as mean ± SD. ns, no significance, *$p < 0.05$, **$p < 0.01$, ***$p < 0.001$.

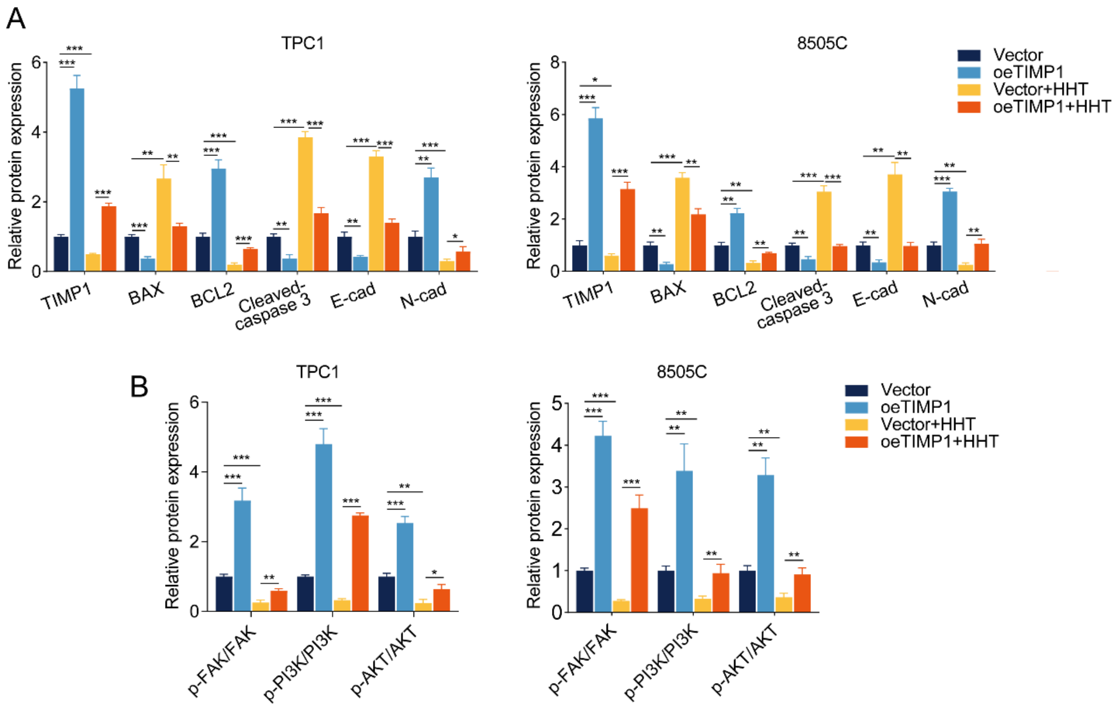

Figure S6. Semi-quantitative analysis of protein expression in Figures 3B and 3J, related to Figure 3. (A) Semi-quantification analysis of apoptosis- and EMT-related protein expression in TPC-1/ oeTIMP1 and 8505C/oeTIMP1 cells with HHT treatment for 48h (Fig. 3B). **(B)** Semi-quantification analysis of FAK, PI3K, AKT, p-FAK, p-PI3K, and p-AKT expression in TPC-1/ oeTIMP1 and 8505C/oeTIMP1 cells with HHT treatment for 48h (Fig. 3J). GAPDH was used as the control. Data are presented as mean ± SD. $^*p < 0.05$, $^{**}p < 0.01$, $^{***}p < 0.001$.

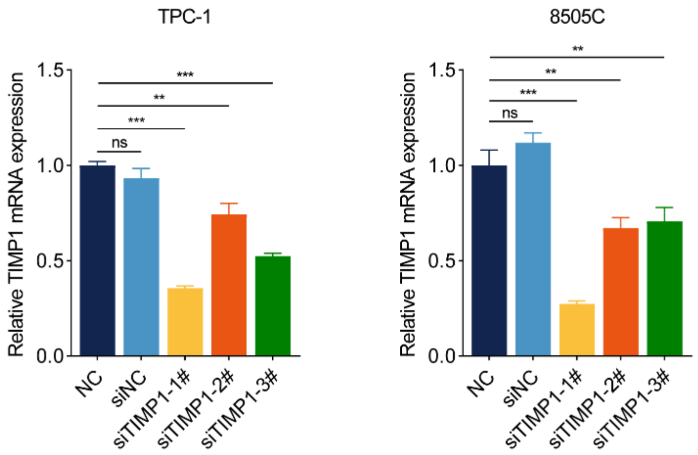

Figure S7. The knockdown efficiency of TIMP1 in TC cells after transfected with TIMP1 siRNA, related to Figure 4. RT-qPCR detected the mRNA level of TIMP1 in TPC-1 and 8505C cells transfected with TIMP1 siRNA. GAPDH was used as the control. Data are presented as mean ± SD. ns, no significance, **$p < 0.01$, ***$p < 0.001$.

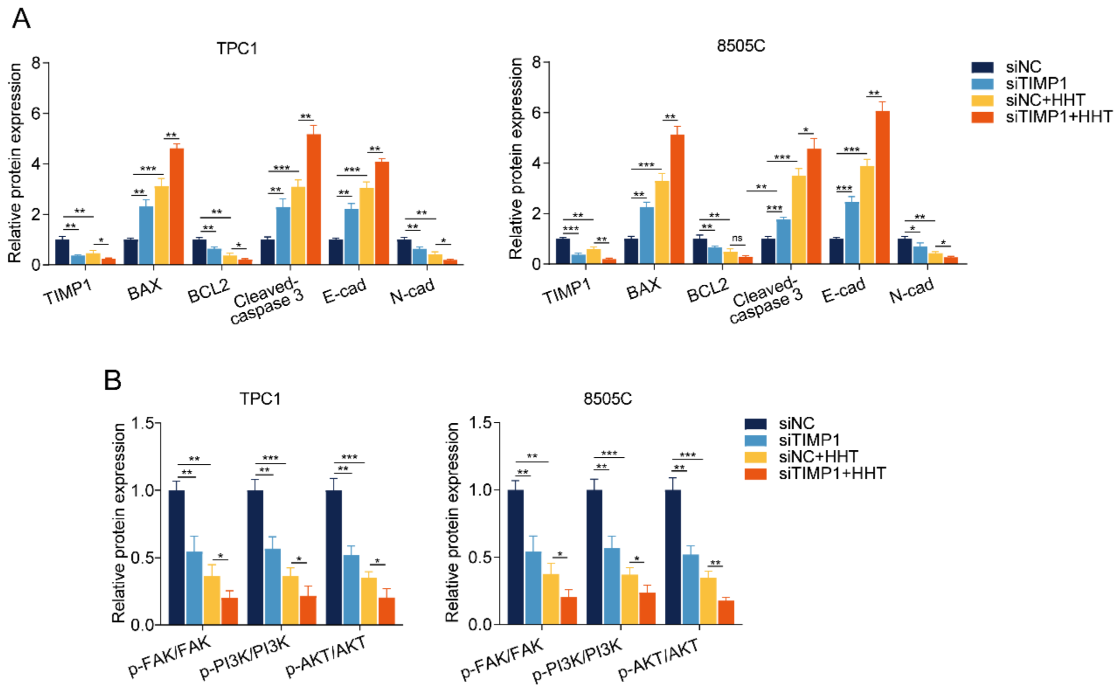

Figure S8. Semi-quantitative analysis of protein expression in Figures 4B and 4J, related to Figure 4. (A) Semi-quantification analysis of apoptosis- and EMT-related proteins in TPC-1/siTIMP1 and 8505C/siTIMP1 cells with HHT treatment for 48h (Fig. 4B). **(B)** Semi-quantification analysis of FAK, PI3K, AKT, p-FAK, p-PI3K, and p-AKT expression in TPC-1/siTIMP1 and 8505C/siTIMP1 cells were treated with HHT treatment for 48h (Fig. 4J). GAPDH was used as the control. Data are presented as mean ± SD. ns, no significance, *$p < 0.05$, **$p < 0.01$, ***$p < 0.001$.

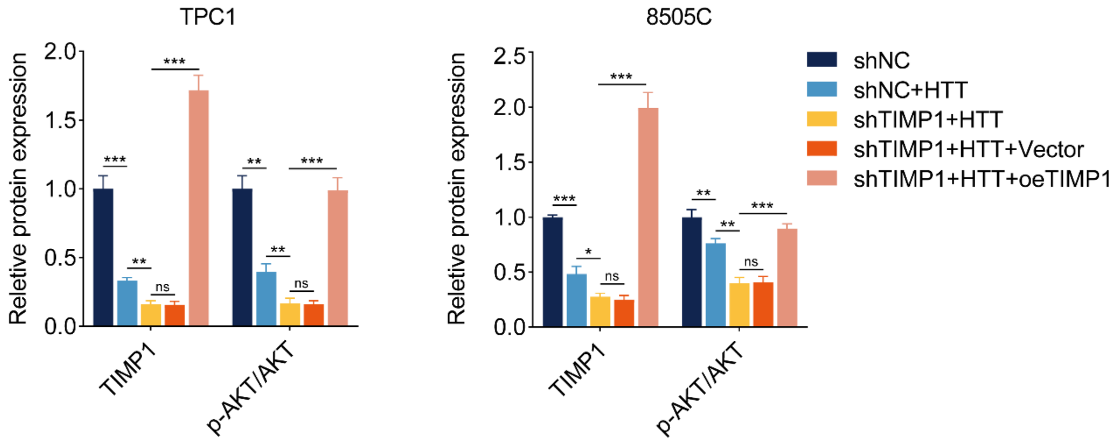

Figure S9. Semi-quantitative analysis of protein expression in Figures 5D, related to Figure 5. Semi-quantification analysis of TIMP1, AKT, and p-AKT expression in TPC-1 and 8505C cells with shTIMP1 and/or oeTIMP1 upon HHT treatment for 48h. GAPDH was used as the control. Data are presented as mean ± SD. ns, no significance, $^{*}p < 0.05$, $^{**}p < 0.01$, $^{***}p < 0.001$.

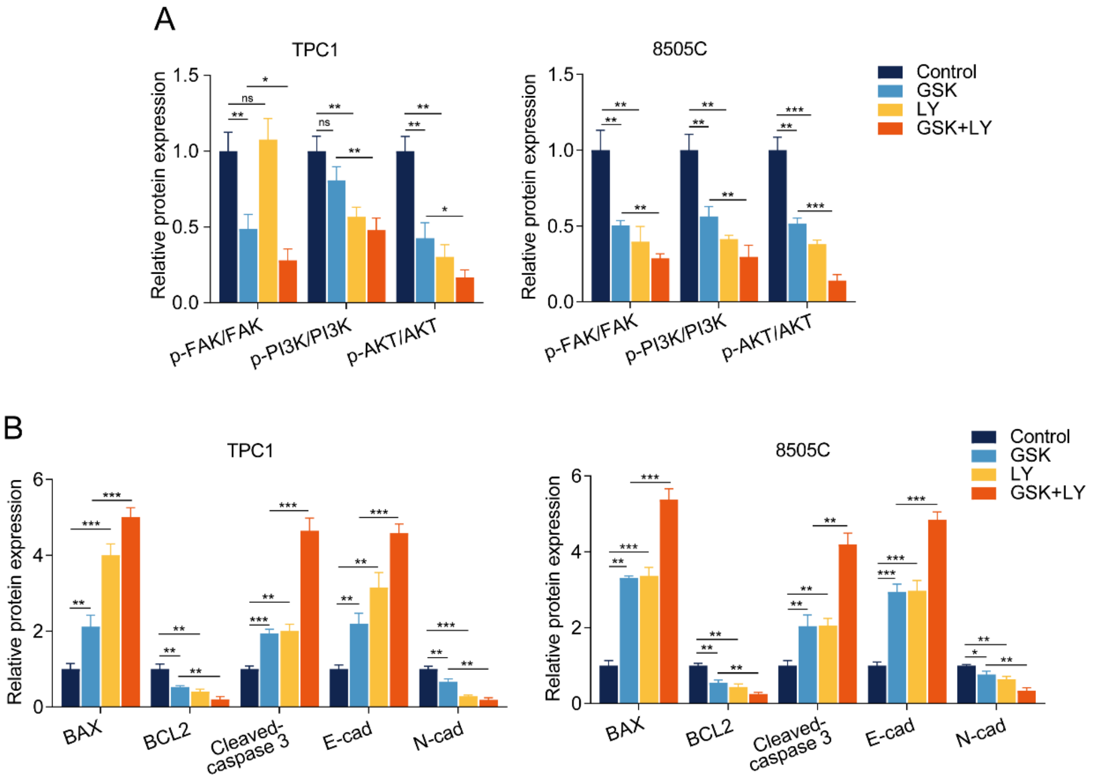

Figure S10. Semi-quantitative analysis of protein expression in Fig. 7A and 7F, related to Figure 7. (A) Semi-quantification analysis of FAK, PI3K, AKT, p-FAK, p-PI3K, and p-AKT expression in TPC-1/ oeTIMP1 and 8505C/oeTIMP1 cells with GSK and/or LY treatment (Fig. 7A). **(B)** Semi-quantification analysis of apoptosis- and EMT-related proteins in TPC-1/ oeTIMP1 and 8505C/oeTIMP1 cells with GSK and/or LY treatment (Fig. 7F). GAPDH was used as the control. Data are presented as mean ± SD. ns, no significance, *p < 0.05, **p < 0.01, ***p < 0.001.

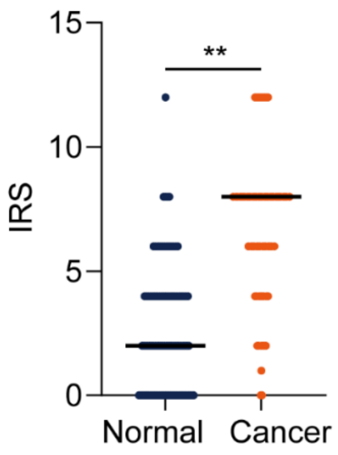

Figure S11. TIMP1 is overexpressed in TC tissues, related to Figure 8. IHC staining detected the TIMP1 expression of normal thyroid tissues and TC tissues. IHC staining score was based on the IRS system. **$p < 0.01$.

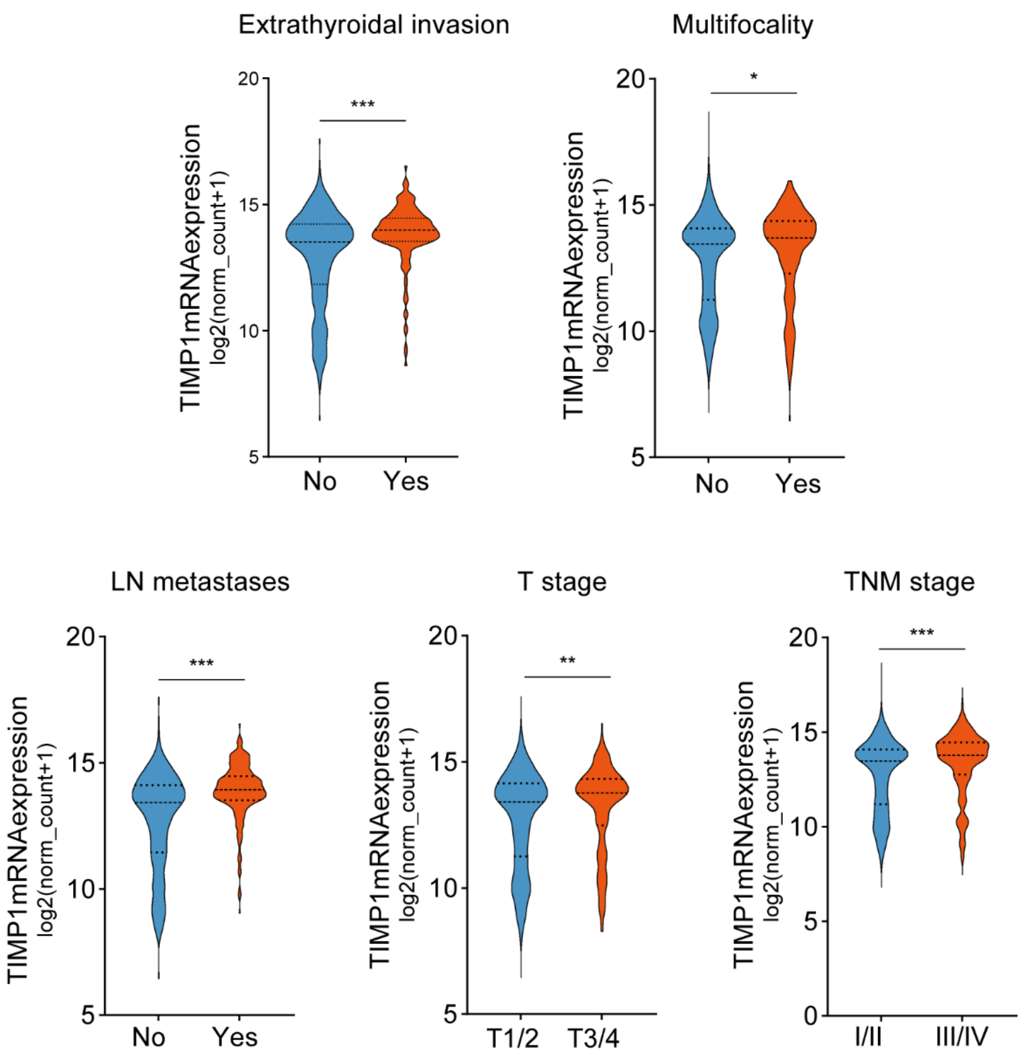

Figure S12. The correlation between high TIMP1 expression and invasive clinical features, related to Figure 8. The high mRNA expression of TIMP1 in TC tissues from the TCGA data was correlated to extrathyroidal invasion, multifocality, lymph node metastases, higher T stage, and higher TNM stage. *$p < 0.05$, **$p < 0.01$, ***$p < 0.001$.

Table S3. The sequence for siRNAs and shRNAs targeting TIMP1, related to STAR Methods

Name	Target sequences (5'→3')
siTIMP1-1#	GATGGACTCTTGCACATCA
siTIMP1-2#	AGATGACCAAGATGTATAA
siTIMP1-3#	GCACAGTGTTTCCCTGTTT
shTIMP1-1#	TGCTGTTGACAGTGAGCGAAAGATGACCAAGATGTATAAAGTTAGTGAAGCCACAGATGTAACTTTATACATCTTGGTCATCTTCTGCCTACTGCCTCGGA
shTIMP1-2#	TGCTGTTGACAGTGAGCGAACACAGTGTTTCCCTGTTTATCTTAGTGAAGCCACAGATGTAAGATAAACAGGGAAACACTGTGTCTGCCTACTGCCTCGGA
shTIMP1-3#	TGCTGTTGACAGTGAGCGAAGGACTCTTGCACATCACTACCTTAGTGAAGCCACAGATGTAAGGTAGTGATGTGCAAGAGTCCTCTGCCTACTGCCTCGGA

Table S4. Primers sequences for RT-qPCR, related to STAR Methods

Primer		Sequences (5'→3')
TIMP1	Forward	AGAGTGTCTGCGGATACTTCC
	Reverse	CCAACAGTGTAGGTCTTGGTG
NIS	Forward	GCGTGGCTCTCTCAGTCAA
	Reverse	GCGTCCATTCCTGAGCTG
GAPDH	Forward	GGAGCGAGATCCCTCCAAAAT
	Reverse	GGCTGTTGTCATACTTCTCATGG

Table S5. Antibodies for Western blotting, related to STAR Methods

Antibody	Source	Cat no.
Bax	proteintech	50599-2-Ig
Bcl2	millipore	AB1722
Cleaved caspase-3	CST	#9664T
E-cadherin	proteintech	20874-1-AP
N-cadherin	proteintech	22018-1-AP
NIS	abcam	ab83816
TIMP1	abcam	ab211926
FAK	CST	#3285
p-FAK	CST	8556s
PI3K	zen bio	R22768
p-PI3K	CST	#4228
AKT	CST	#4691
p-AKT	CST	#4060S
GAPDH	CST	#5174
